# Association of oral frailty with falls in long-term care residents

**DOI:** 10.1007/s41999-024-01088-6

**Published:** 2024-11-06

**Authors:** Taija Puranen, Kaija Hiltunen, Kaisu H. Pitkälä, Hanna-Maria Roitto, Päivi Mäntylä, Riitta K. T. Saarela

**Affiliations:** 1 Department of General Practice and Primary Health Care, University of Helsinki, Helsinki, Finland; 2 Department of Oral and Maxillofacial Diseases, University of Helsinki, Helsinki, Finland; 3 Helsinki University Hospital, Unit of Primary Health Care, Helsinki, Finland; 4 Department of Geriatrics, Helsinki University Hospital and University of Helsinki, Helsinki, Finland; 5 Finnish Institute for Health and Welfare, Population Health Unit, Helsinki, Finland; 6 Institute of Dentistry, University of Eastern Finland, Kuopio, Finland; 7 Kuopio University Hospital, Oral and Maxillofacial Diseases, Kuopio, Finland; 8 City of Helsinki, Social Services, Health Care and Rescue Services Division, Oral Health Care, City of Helsinki, Helsinki, Finland

**Keywords:** Oral frailty, Falls, Frailty, Long-term care

## Abstract

**Purpose:**

We investigated the relationship between oral frailty (OFr) and falls among long-term care residents.

**Methods:**

Residents (N = 254, mean age 84 y, 79% women) participated in this longitudinal cohort study in 2018–2021. OFr was defined by six signs. Demographics, diagnoses, and medications were retrieved from the medical records. Frailty phenotype, sarcopenia and disability were assessed. Number of falls was collected from medical records over 12 months following baseline assessment.

**Results:**

Of participants, 35% fell during follow-up. Of the fallers, 28% were bedridden or needed a wheelchair; among non-fallers, this figure was 73%. In logistic regression analysis after adjusting for age, sex, BMI, stroke, dementia, diabetes, coronary heart disease, number of medications, mobility, walking speed and sarcopenia, OFr did not predict falls (OR for severe OFr 0.13 (95% CI 0.01—1.27). Male sex predicted falls.

**Conclusions:**

No association was found between severe OFr and falls over a 12-month follow-up.

## Key summary points

**Aim:**

We investigated the relationship between oral frailty and falls among long-term care residents.

**Findings:**

Severity of oral frailty did not predict falls. Non-fallers were more often bedridden or needed a wheelchair and were dependent on others for activities of daily living. Male sex predicted falls.

**Message:**

The association between oral frailty and falls appears to differ between home-dwelling older adults and long-term-care residents. Mobility and dependency in activities of daily living may play a role as mediating factors.

## Background

Among community-dwelling older adults, oral frailty (OFr) has been associated with poor gait performance, which is a known risk factor for falls [1]. Kamide and colleagues [2] investigated the relationship between OFr and falls over one year in community-dwelling older people. In their observational study, they found that OFr was directly associated with falls in older people who were independent in their instrumental activities of daily living (IADL) [2]. A recent meta-analysis suggested that incidence of falls is high (43%) among nursing home residents, and the risk factors with a strong association with falls included fall history, impaired performance in activities of daily life, insomnia, and depression [3].

Oral health problems are associated with disability [4]. At present, there is no solid consensus on the definition and measurement of OFr [5, 6]. In long-term care (LTC), Hiltunen and colleagues [7] suggested that six oral signs (1. dry mouth; 2. food residue on surface of teeth, mucosa, or dentures; 3. unclear speech; 4. inability to keep mouth open; 5. oral pain; 6. diet of pureed or soft food) may be used to determine OFr syndrome. That study found a significant linear relationship between OFr severity and Fried’s frailty phenotype; participants with more OFr signs suffered more often from frailty, dementia, and malnutrition and more commonly required help with daily activities than those with fewer signs [7]. Furthermore, OFr has been associated with reduced health-related quality of life and mortality [8], as well as with sarcopenia and functional disability [9].

Inspired by Kamide and colleagues’ study [2], we aimed to investigate the relationship between OFr and falls, and associated factors in the LTC residents.

## Methods

This study is part of a larger project exploring the nutritional status and quality of nutritional care among LTC residents in Helsinki, Finland [10]. Trained nurses assessed 550 residents in LTC. They collected information on demographics and mobility (bed- or chair-bound; able to get out of bed / chair but does not go out; goes out) and dependence in activities of daily living (ADL) based on Clinical Dementia Rating (CDR) personal care class ≥ 2. Number of medications, diagnoses, height, and weight were retrieved from medical records, and the Charlson comorbidity index (CCI) and body mass index (BMI) were calculated. Frailty status was defined according to Fried’s frailty phenotype [11], and sarcopenia with the European definition [12]. Comprehensive clinical oral examinations were performed on 254 LTC residents (FINORAL study). OFr was defined by six signs as described elsewhere [7]. The sum of the OFr signs determined the severity of OFr: no/mild OFr (0 or 1 sign); moderate OFr (2–4 signs); severe OFr (5–6 signs) [7]. Data on falls were collected from medical records during the 12-month follow-up.

Our study population was divided into fallers and non-fallers. The characteristics were compared with X^2^-test for categorical variables and Mann–Whitney test for continuous variables. Multivariate logistic regression analysis was performed to analyze the association between falls and OFr adjusted for age, sex, BMI, stroke, dementia, diabetes, coronary heart disease, number of medications, mobility, walking speed and sarcopenia. Statistical analysis was performed using NCSS statistical software.

The Ethics Committee of Helsinki University Hospital and the City of Helsinki approved the study protocol. Written informed consent was obtained from the participants or in case of moderate–severe dementia from their closest proxies.

## Results

The mean age of participants was 84 years and 79% were women. Of the study participants, 35% (n = 89) fell at least once during the 12-month follow-up. The fallers and non-fallers (n = 165) did not differ with respect to age, BMI, CCI, dementia or sarcopenia. The fallers fell a mean of 3.3 times during the 12-month follow-up. Of the fallers, 28% were bedridden or needed a wheelchair; the corresponding figure among non-fallers was 73%. Dependence in ADL was more common among non-fallers than fallers (94% vs. 79%). In addition, fallers more often had coronary heart disease, (23% vs. 11%) and were administered a higher number of medications (9.3 vs. 8.5). A larger proportion of non-fallers were females (84% vs.71%), more often had stroke (29% vs. 17%), were frail (75% vs. 46%), and had a walking speed of < 0.8 m/sec (95% vs. 85%). Of the fallers, 24% had no/mild OFr, 70% moderate OFr, and 7% severe OFr, whereas the respective figures for non-fallers were 13%, 66%, and 21% (Table 1).

**Table 1:** Participants´ characteristics and comparison between fallers and non-fallers

	Fallers N = 89 (35%)	Non-fallers N = 165 (65%)	P for linearity
Age, years, mean (SD)	84 (8)	84 (8)	0.68
Women, n (%)	63 (71)	138 (84)	0.016
Dependence in ADL, n (%)	70 (79)	155 (94)	< 0.001
BMI, mean (SD)	27.0 (52)	25.8 (5.5)	0.10
Charlson comorbidity index, mean (SD)	2.0 (1.3)	2.6 (2.7)	0.36
Diabetes, n (%)	20 (23)	26 (16)	0.18
Coronary heart disease, n (%)	20 (23)	18 (11)	0.014
Stroke, n (%)	15 (17)	47 (29)	0.040
Dementia, n (%)	69 (78)	121 (73)	0.46
Number of medications, mean (SD)	9.3 (3.2)	8.5 (3.9)	0.029
Number of falls, mean (SD)	3.3 (3.5)	0 (0)	< 0.001
Bedridden or needs wheelchair, n (%)	25 (28)	120 (73)	< 0.001
Fried’s frailty phenotype, n (%)			< 0.001
Robust	1 (1)	1 (1)	
Prefrail	47 (53)	41 (25)	
Frail	41 (46)	123 (75)	
Walking speed < 0.8 m/sec, n (%)	76 (85)	157 (95)	0.007
Sarcopenia, n (%)	1/56 (2)	8/93 (9)	0.091
Oral frailty categories, n (%)			0.004
I (no/mild 0–1 signs)	21 (24)	22 (13)	
II (moderate 2–4 signs)	62 (70)	108 (66)	
III (severe 5–6 signs)	6 (7)	35 (21)	

ADL = activities in daily living; dependency defined as personal care class ≥ 2 in Clinical Dementia Rating; BMI = body mass index; Fried’s Frailty phenotype (11); Sarcopenia defined according to European Working Group on Sarcopenia (12)

In logistic regression analysis adjusting for various confounding factors and using no/mild OFr as a reference (1.0), neither moderate OFr (OR 1.00; 95% CI 0.39—2.60) or severe OFr predicted falls (OR 0.13; 95% CI 0.01–1.27). Being bedridden or needing a wheelchair was protective against falls (OR 0.24; 95% CI 0.10—0.56). Male sex did predict falls (OR 3.61; 95% CI 1.37–9.50), but no other factors were associated with falls.

## Discussion

Our main finding was that OFr did not predict falls. Of the residents, 35% fell during the 12-month follow-up. Non-fallers more often were bedridden or needed a wheelchair than fallers. Of confounding factors, only being bedridden or needing a wheelchair was protective against falls, whereas male sex predicted falls.

The proportion of fallers (35%) in our study was slightly higher than in the study of Castaldo and colleagues [13], who reported that 27% of LTC residents fell during one year, but lower than in the study of Cameron and the colleagues [14], who reported 56% of LTC residents to fall during a six-month follow-up. Moderate or severe OFr in our study were not associated with falls, which is in contrary to the finding of Kamide and colleagues [2] in home-dwelling older adults. Several differences between our study and that of Kamide and colleagues may explain the conflicting findings. In our clinical study, the participants were LTC residents, thus, older and more often frail with more disabilities. Especially in the non-fallers’ group, participants were mostly bedridden or needed a wheelchair, which obviously reduces the likelihood of falls and may explain our finding. In line with our study, Castaldo and colleagues [13] noted that higher autonomy in ADL was associated with an increased number of falls. In contrast, Shao and colleagues [3] reported impaired ADL a strong risk factor for falls. In our study, fallers used more medications than non-fallers, consistent with a previous report [3]. We found no difference between fallers and non-fallers in dementia in contrast to an earlier report [3]. In line with our study, male sex was associated with falls in nursing home setting [3, 14], but not among home-dwelling older adults [2].

To define OFr, we used six signs previously used in three studies [7, 8, 15]. In the LTC setting with residents needing constant care and the majority having dementia, questionnaires or complicated assessments are not practical to accomplish as suggested for example by Morley [5] with D-E-N-T-A-L or EAT-10 questionnaires and Tanaka and colleagues [6] with the Oral Frailty Index-8 questionnaire (OFI-8). The signs selected to detect OFr in our study are easy to use bedside in LTC, enabling nurses to assess the residents [7, 8, 15]. OFr is an important indicator since it is associated with frailty phenotype and lower health-related quality of life and mortality [7, 8]. Therefore, a simple method is needed to determine OFr in LTC residents that does not depend on the individual, her/his health, dependence on ADL, living environment or availability of dentists. Screening for falls in LTC is important, as Castaldo and colleagues [13] reported that nearly half of fallers had a fall-related injury. In addition, further research is warranted to identify fall risk factors and to develop and implement multifactorial interventions to prevent falls.

Strengths of this study are the well-validated and widely used assessment tools and a structured questionnaire validated in previous nutritional studies in Helsinki in 2003–2011 [10]. A limitation is the cross-sectional nature of the study baseline assessments, although we had a longitudinal design concerning falls. It is, however, challenging to draw conclusions about the causal relationship between OFr and falls. The advantage of the current OFr definition is that it is easy to employ in clinical practice, but it has been used only in three studies before and has solely been tested in the LTC environment [7, 8, 15]. For now, our measure of OFr with six signs has shown concurrent validity with frailty phenotype and health related quality of life and prognostic validity with mortality [7, 8]. It did not predict falls in this population and clearly needs further testing in other types of populations. Studies are currently underway. In addition, in oral examination, as the participants were lying on their bed or sitting on a chair, the accuracy of the examination does not correspond to that of examination performed in a dentist’s office with better visibility [7, 8, 15].

## Conclusion

Based on our new method of assessing OFr the relationship between falls and OFr appears to differ between community-dwelling older adults and LTC residents. Poor mobility and protection of those needing help in their transferring may have a role as mediating factors. The methods of assessing OFr and their associations need to be tested further in both research and clinical practice.
